# Genome-Wide Association Study of Susceptibility to Infection by *Mycobacterium avium* Subspecies *paratuberculosis* in Holstein Cattle

**DOI:** 10.1371/journal.pone.0111704

**Published:** 2014-12-04

**Authors:** Fazli Alpay, Yalda Zare, Mamat H. Kamalludin, Xixia Huang, Xianwei Shi, George E. Shook, Michael T. Collins, Brian W. Kirkpatrick

**Affiliations:** 1 Department of Animal Science, Faculty of Veterinary Medicine, Uludag University, Bursa, 16059, Turkey; 2 Department of Animal Sciences, University of Wisconsin-Madison, Madison, Wisconsin, 53706, United States of America; 3 Department of Animal Science, Faculty of Agriculture, Universiti Putra, UPM Serdang, Selangor, Malaysia; 4 College of Animal Science, Xinjiang Agricultural University, Urumqi, Xinjiang, China; 5 Department of Dairy Science, University of Wisconsin-Madison, Madison, Wisconsin, 53706, United States of America; 6 Department of Pathobiological Science, School of Veterinary Medicine, University of Wisconsin-Madison, Madison, Wisconsin, 53706, United States of America; University of New South Wales, Australia

## Abstract

Paratuberculosis, or Johne's disease, is a chronic, granulomatous, gastrointestinal tract disease of cattle and other ruminants caused by the bacterium *Mycobacterium avium*, subspecies *paratuberculosis* (*MAP*). Control of Johne's disease is based on programs of testing and culling animals positive for infection with *MAP* while concurrently modifying management to reduce the likelihood of infection. The current study is motivated by the hypothesis that genetic variation in host susceptibility to *MAP* infection can be dissected and quantifiable associations with genetic markers identified. For this purpose, a case-control, genome-wide association study was conducted using US Holstein cattle phenotyped for *MAP* infection using a serum ELISA and/or fecal culture test. Cases included cows positive for either serum ELISA, fecal culture or both. Controls consisted of animals negative for the serum ELISA test or both serum ELISA and fecal culture when both were available. Controls were matched by herd and proximal birth date with cases. A total of 856 cows (451 cases and 405 controls) were used in initial discovery analyses, and an additional 263 cows (159 cases and 104 controls) from the same herds were used as a validation data set. Data were analyzed in a single marker analysis controlling for relatedness of individuals (GRAMMAR-GC) and also in a Bayesian analysis in which multiple marker effects were estimated simultaneously (GenSel). For the latter, effects of non-overlapping 1 Mb marker windows across the genome were estimated. Results from the two discovery analyses were generally concordant; however, discovery results were generally not well supported in analysis of the validation data set. A combined analysis of discovery and validation data sets provided strongest support for SNPs and 1 Mb windows on chromosomes 1, 2, 6, 7, 17 and 29.

## Introduction

Paratuberculosis, or Johne's disease, is a chronic, granulomatous, gastrointestinal tract disease of cattle and other ruminants caused by the bacterium *Mycobacterium avium* subspecies *paratuberculosis* (*MAP*). The clinical signs of disease in cattle are pipestream diarrhea, weight loss, edema due to hypoproteinemia caused by protein-losing enteropathy [1]. Calves less than 6 months of age are generally considered to be at the greatest risk of becoming infected with MAP [2], but clinical signs of infection usually do not appear until second or third lactation [3]. Even if not showing clinical signs of disease, *MAP* test-positive cows produce less milk and are culled earlier in their productive life [2].

The disease occurs worldwide in dairy cattle and other ruminants. Control programs for paratuberculosis have been established in some nations including Australia [4], Norway [5], Iceland [6], Japan [7], the Netherlands [8] and the United States [9]. The reported herd-based prevalence of *MAP* infection varies between European countries and is greater than 60% in some regions according to a recent review [10]. Recent estimates suggest that 68% of US dairy herds [11] and 7.9% of US beef herds have infected animals [12]. After accounting for assay sensitivity, true prevelance at a herd level has been estimated to exceed 90% [13]. The economic impact of paratuberculosis on the US dairy industry has been estimated to be from US $200 million to $1.5 billion annually [14], [15]. An additional concern is the potential zoonotic role of *MAP* in Crohn's disease in humans, which at the current time remains uncertain [16].

There currently is no cure for Johne's disease, and vaccination is problematic. Routine testing combined with culling currently provide the best opportunity for controlling the disease. Knowledge concerning genetics of susceptibility to *MAP* infection can contribute to disease control programs by facilitating genetic selection for a less susceptible population to reduce incidence of infection in the future. The opportunity for genetic improvement in susceptibility to infection is evidenced by estimates of heritability of *MAP* infection in dairy cattle ranging from 0.03 to 0.28 [17], [18], [19], [20], [21], [22], [23] and differences among dairy sires in prevalence of *MAP* infection of their daughters [19], [24],[25].

Genetic factors affect susceptibility to common diseases, and genome wide association studies (GWAS) provide a tool for identifying these genetic factors [26], [27], [28]. The objectives of this study were to identify genomic regions associated with susceptibility to *MAP* infection in Holstein cattle and develop tools for genomic selection for lesser susceptibility to *MAP* infection.

## Materials and Methods

The University of Wisconsin-Madison College of Agricultural and Life Sciences Animal Care and Use Committee approved this research.

### Animal resources

Two resource populations of approximately 5,000 Holstein cows each were used to identify genomic regions associated with susceptibility to infection by *MAP*. Population 1 consisted primarily of twelve paternal half-sib families of daughters of sires heavily used within the US Holstein population. Collection of these samples has been described previously (Gonda et al. 2006). Samples were obtained mainly during 2001 to 2003 from 300 herds from across the US with Wisconsin and California herds together accounting for approximately 48% of the total samples. Population 2 consisted of cows from six commercial Holstein herds in Wisconsin that were cooperators in a Johne's disease control project. Blood samples for disease testing and DNA extraction were obtained from all cows in these herds over a period of 15 months in 2006-7 [29].

Phenotype for *MAP* infection in Population 1 was based on both fecal culture of *MAP* and evidence of antibody titer to *MAP* as based on a serum ELISA test. Fecal culture for *MAP* was measured over 12 weeks using a radiometric BACTEC method [30]. Regarding ELISA testing, samples from Population 1 had been originally tested using the IDEXX ELISA, but were re-tested using a more recently developed ELISA (JTC-ELISA) with higher sensitivity (Shin et al., 2008). Phenotypes for Population 2 were based on ELISA results, also with the higher sensitivity test. Optical density values of the serum from project animals and from positive and negative controls were converted to sample to positive ratios (S/P). Based on the value of S/P ratio, results of ELISA tests were categorized as negative (0 to 0.09), suspect (0.10 to 0.24), low positive (0.25 to 0.39), positive (0.40 to 0.99), and strong positive (≧1.00) as suggested by [31]. Animals categorized as strong-positive, positive or low-positive were all considered to be ELISA-positive. The sensitivity and specificity of this test has been estimated to be approximately 30% and>99%, respectively relative to fecal culture [32]. Cases were defined as animals positive to either ELISA or fecal culture testing, and controls were those negative for ELISA and negative for the fecal culture test (if available), matched for both herd and nearest birth date.

### Genotyping, Quality Control and Imputation

Samples from both populations were genotyped with the Illumina Bovine SNP50 BeadChip for discovery analysis and Illumina BovineLD BeadChip for validation analysis. A combined total of 856 of 890 samples (451 cases and 405 controls) from Populations 1 and 2 were successfully genotyped by either Illumina, Inc. (San Diego, CA), GeneSeek Inc. (Lincoln, NE) or the University of Wisconsin Biotechnology Center (UWBC). These samples are referred to herein as the discovery data. The initial genotyping by Illumina and GeneSeek [33] employed version 1 of the SNP50 BeadChip, while subsequent genotyping employed version 2. Initially, only cases were genotyped for use in a case-reference analysis (Kirkpatrick et al., 2010). The genotyping of case and control samples with two different versions of the 50 K bead chip created a potentially problematic scenario from the standpoint that discrepancies in genotyping between chip versions would potentially lead to false positive results. Twenty-two samples were genotyped using both versions of the chip permitting identification of a subset of SNPs that were discrepantly genotyped (<95% concordance) between the two chip versions; these were omitted from subsequent analyses (12,504 of the 51,552 SNPs in common between chips). Additionally, animals with fewer than 90% successfully scored genotypes and SNPs that were successfully scored for fewer than 95% of the samples in either of the two resource populations were removed prior to statistical analyses. Quality control (QC) was implemented on SNPs using PLINK [34]. SNPs with unknown genomic location, not in Hardy-Weinberg equilibrium (p<10^−6^) or with minor allele frequencies below 0.02 were also not included in statistical analyses. After exclusion for these various reasons, a total of 33,484 SNPs remained.

For validation analyses, 103 samples from Populations 1 and 2 (negative for *MAP* infection based on ELISA test results and fecal culture test results if available) were genotyped by DNA Landmarks, Inc. (Québec, Canada), using the BovineLD BeadChip. These samples plus 160 samples from Population 1 and 2 (positive for *MAP* infection based on either ELISA or fecal culture) previously genotyped with the SNP50 BeadChip and masked for all but the 6,844 SNPs in common between the 50 K and LD BeadChips constituted the validation data set.

Data sets from SNP50 BeadChips and BovineLD BeadChip were merged for imputation purposes. Imputation of genotypes for missing or untyped markers was performed using BEAGLE version 3.3 [35]. Imputation was conducted using all BEAGLE default options.

### Genome-wide Association Analyses

Association analysis under an additive model was performed using the GRAMMAR-GC approach (Genome-wide association using Mixed Model and Regression) as implemented within the GenABEL package [36] for R [37]. First, a polygenic analysis was conducted using a genomic kinship matrix based on SNP genotypes to account for relationship between individuals [38]. Residuals from the polygenic analysis were then used as dependent, quantitative variables in single marker, linear regression analyses with significance of marker effects determined as previously described [39]. A false discovery rate approach was used to identify the most significant SNP using a threshold of 0.5 meaning a 50% or greater probability that the identified association was not a false positive [40].

A second GWAS analysis was implemented with a Bayes C model averaging approach using the GenSel program [41] The Bayes C method is derived from the Bayes B approach [42]. Bayes C uses a common variance for SNP effects that is reliably estimated from the SNP data and is less sensitive to the priors than is Bayes B [43]. The phenotypic distribution for *MAP* infection was discrete, and consequently the data were analyzed using a categorical threshold analysis in GenSel. Individual SNP effects were estimated from a mixture model with the value of π set at 0.999, meaning that one out of a thousand SNPs were included in the model in any particular iteration of the Bayesian analysis and given a non-zero effect estimate. This high π value has been shown to give faster convergence in the model averaging procedures [44], and focuses the results on the most significant SNPs while including every SNP in some small proportion of the models. A total of 41,000 iterations in a Monte Carlo Markov Chain (MCMC) with a burn-in of 1000 iterations were run for the analyses. Results from this analysis included posterior distributions for the effects of each of the 33,484 markers, adjusted for the portfolio of all the other fitted marker effects in the model in each iteration of the chain.

Effects of an underlying (ungenotyped) causative locus may be divided between multiple SNP loci in the Bayesian analysis. To more effectively identify genomic regions harboring quantitative trait loci (QTL), association of groups of consecutive SNPs in 1 Mb windows were analyzed using the windowsBV option in GenSel. Windows were defined as spanning consecutive, non-overlapping 1 Mb regions across the genome. Genetic variance for a window as a proportion of total genetic variance across the genome was used to identify the most informative regions. Posterior probability of inclusion (PPI), which is the proportion of samples of the Markov chain in which a given SNP window was included in the model with a non-zero effect was calculated and those exceeding an arbitrary threshold of PPI>0.20 are reported. For both GenABLE and GenSel analyses, the analysis was applied to the 50 K discovery data, the validation data imputed to 50 K and combined discovery and validation data sets.

### Cross-validation Analysis

The accuracy of genomic predictions was evaluated by pooling estimates using a 5-fold cross-validation strategy. Genotyped animals were divided randomly (within cases and controls) into five exclusive groups. In each training analysis, four of the five groups were combined to comprise a training data set to estimate marker effects using Bayes C analysis. Results from this analysis were then used to predict genomic values of individuals from the omitted group (testing set) using the GenSel program. Additionally, a sixth analysis was performed in which the discovery data comprised the training data set and the validation data comprised the testing data set.

Prediction efficacy was evaluated by Receiver Operating Characteristic (ROC) analysis [45] using the ROCR package [46] in R. A pair of observations with different observed responses (case vs. control) was concordant if the observation with the lower ordered response value had a lower predicted score than the observation with the higher ordered response value. All possible pairs within each testing group were evaluated in this manner. This analysis was repeated for all five of the cross-validation training and testing combinations as well as the discovery and validation training and testing sets. To provide a context for results of these analyses, a simulation was performed in which 1,000 records were generated corresponding to a normally distributed liability for *MAP* infection with h^2^ = 0.10. A threshold was applied to achieve frequencies of cases corresponding to 10%. Probability of correct classification based on the known, simulated genetic effects was then calculated in the same manner as above for the real data with the simulation repeated 1,000 times.

## Results

Regarding diagnostic evaluations, examination of a multi-dimensional scaling plot (Figure S1) for the combined discovery and validation data revealed no evidence of population substructure (ie. no discrete clusters). Examination of a quantile-quantile plot for results from the GRAMMAR-GC analysis of combined discovery and validation data was consistent with little departure from the null hypothesis of no association of infection test result with SNP for most SNPs and relatively few SNPs having significant effects (Figure S2).

**Figure 1:**
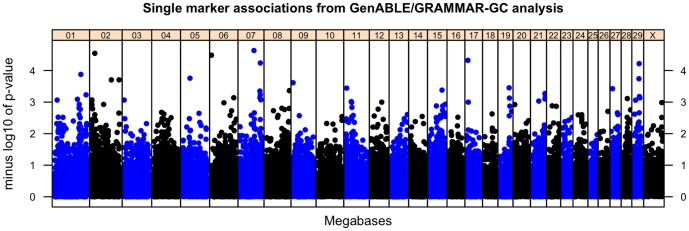
Manhattan plot for single marker (GRAMMAR-GC/GenABLE) analysis of combined discovery and validation data sets. Each dot represents the results from the test of association for a single SNP. Minus log_10_ of the p-value is indicated on the y-axis and map location of the SNP is indicated on the x-axis.

Markers significantly associated with *MAP* infection phenotype (p<5×10^−5^ or false discovery rate <0.5) are presented in Table 1 ordered by significance from the analysis of combined discovery and validation data sets. Single marker GRAMMAR-GC analysis of the discovery data identified two SNPs with significance at p<5×10^−5^ on chromosomes 15 and 7 (Table 1). Neither of these SNP associations were successfully replicated in analysis of the validation data set (p>0.10 and effect estimate of opposite sign). When discovery and validation data were combined and analyzed in a joint analysis, a total of five SNPs had false discovery rates less than 0.5 with nominal p-values ranging from 2.84×10^−5^ to 6.06×10^−5^. Locations of these SNPs were on chromosomes 2, 6, 7, 17 and 29 (Figure 1, Table 1).

**Table 1:** SNP associations from GRAMMAR-GC analysis.

					Discovery			Validation		Combined		
SNP	BTA	bp	alleles	MAF	nominal p-value	FDR^1^	effect ± SE	nominal p-value	effect ± SE	nominal p-value	FDR^1^	effect ± SE
ARS-BFGL-NGS-7756	7	70,988,849	G/A	0.47	3.53×10^−5^	0.59	0.088±0.022	0.14	−0.056±0.038	2.32×10^−5^	0.78	0.078±0.019
ARS-BFGL-NGS-36375	2	15,709,188	A/G	0.28	2.16×10^−2^	1.03	0.054±0.024	3.08×10^−6^	0.194±0.041	2.84×10^−5^	0.48	0.086±0.021
ARS-BFGL-NGS-43717	17	9,392,845	A/G	0.36	9.79×10^−4^	0.89	0.070±0.022	0.02	0.093±0.039	4.86×10^−5^	0.35	0.076±0.019
ARS-BFGL-NGS-2069	6	526,736	G/A	0.23	1.39×10^−4^	1.16	0.085±0.023	0.08	0.061±0.035	5.35×10^−5^	0.37	0.078±0.019
Hapmap38264-BTA-96587	7	102,398,654	C/A	0.46	5.31×10^−4^	0.89	0.074±0.022	0.04	0.077±0.038	5.78×10^−5^	0.39	0.075±0.019
ARS-BFGL-NGS-12309	29	32,671,085	G/A	0.45	2.85×10^−4^	0.96	−0.077±0.022	0.04	0.078±0.038	6.06×10^−5^	0.34	−0.074±0.019
ARS-BFGL-NGS-110386	15	66,653,797	G/A	0.22	1.62×10^−5^	0.54	0.110±0.027	0.59	−0.024±0.045	4.18×10^−4^	0.87	0.079±0.023

1 False discovery rate.

**Figure 2:**
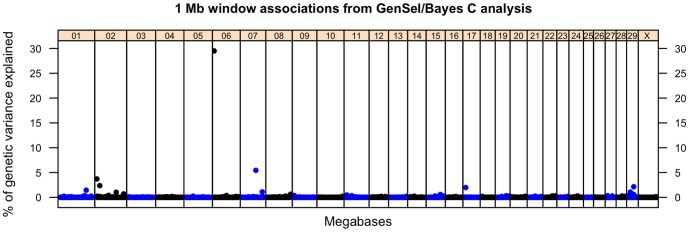
Manhattan plot for 1 Mb window (Bayes C/GenSel) analysis of combined discovery and validation data sets. Each dot represents the percent of genetic variance explained by multiple SNPs within a 1 Mb window. Percent of variance is indicated on the y-axis and map location of the SNP is indicated on the x-axis.

Markers accounting for the greatest proportion of genetic variation in *MAP* infection phenotype in the Bayes C analysis are presented in Table 2 ordered by the percent of variance explained in the analysis of combined discovery and validation data sets. Bayes C analysis of the discovery data identified three 1 Mb windows which were included in the model in more than 20% of the iterations (PPI>0.20) with locations on chromosomes 7, 8 and 15 (Figure 2). The windows on chromosomes 7 and 15 correspond to the location of most significant SNPs from the GRAMMAR-GC analysis of the discovery data. As with the GRAMMAR-GC analysis, addition of the validation did not build support for the chromosome 15 window, and the decrease in support for the chromosome 8 window was even greater. Bayes C analysis of the combined discovery and validation data with GenSel identified 1 Mb windows on chromosomes 1, 2, 6, 7, 17 and 29 that exceeded a PPI of 0.20 (Table 2, Figure 2). For five of the seven most significant windows there was correspondence between window location and locations of individually significant loci from the GRAMMAR-GC analysis (Table 1). The two exceptions were windows at 1–2 Mb on BTA2 and 128–129 Mb on BTA1 without SNPs among the most significant listed in Table 1. Conversely, a significant SNP at 102.4 Mb on BTA7 was without a corresponding significant window from the Bayes C analysis. In both cases the missing SNP or window fell just below the threshold applied for inclusion in Tables 1 and 2, so correspondence between results of the two analytical methods was high.

**Table 2:** Most significant 1 Mb windows from Bayes C analysis of discovery and combined discovery and validation data.

			Discovery		Combined Discovery and Validation	
BTA	Starting location (bp)	Number of SNPs in 1 Mb window	Percent of total SNP variance	p>0^1^	Percent of total SNP variance	p>0^1^
6	202,769	10	1.26	0.12	29.50	0.92
7	70,299,314	9	4.63	0.29	5.44	0.54
2	1,039,834	21	0.37	0.06	3.71	0.47
2	15,001,586	19	0.05	0.02	2.36	0.29
29	32,033,056	16	0.52	0.06	2.14	0.24
17	9,027,765	18	0.32	0.05	1.98	0.24
1	128,031,876	19	0.48	0.07	1.44	0.26
15	66,042,287	14	2.93	0.25	0.58	0.08
8	113,012,644	8	2.87	0.22	0.04	0.01

1 Proportion of iterations in which the window accounted for a proportion of genetic variation greater than zero.

**Figure 3:**
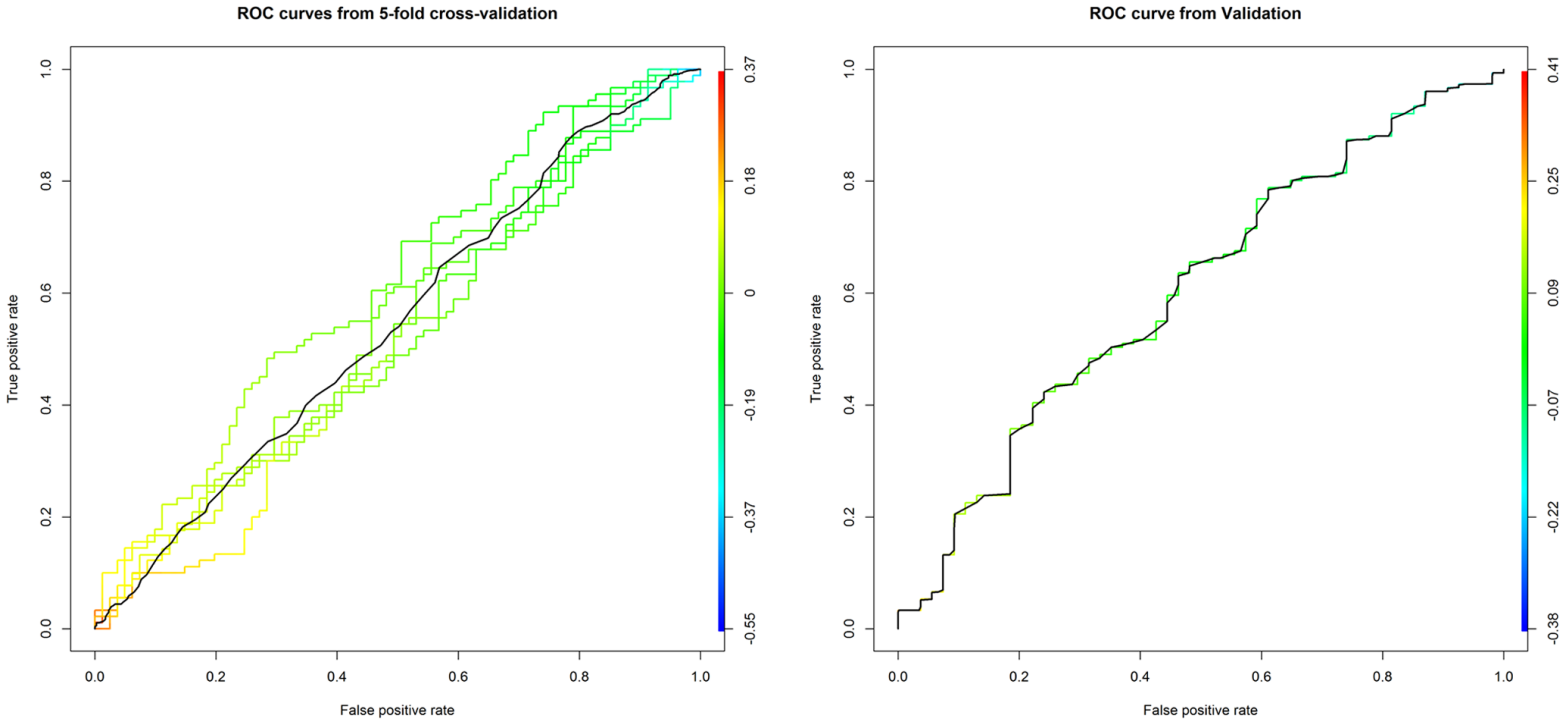
Receiver Operating Characteristics (ROC) curve for five-fold cross-validation and for application of results from discovery data to validation data. Models were developed using a Bayes C analysis implemented in GenSel. Each curve represents one model, with the black line in the figure on the left representing the average of the five-fold cross-validations. Area under the ROC curve is equivalent to the probability that the classifier will rank a randomly chosen positive instance higher than a randomly chosen negative instance. A diagonal line from the lower left to upper right corner would represent a model with no predictive ability.

The ROC curve (Figure 3) of the cross-validation analysis yielded a low probability of correct classification of cases and controls based on genomic prediction. Probabilities ranged from 0.50 to 0.61 with an average of 0.55. Probability of correct classification was marginally higher for the discovery data prediction applied to the validation data, with a value of 0.60. By contrast, the probability of correct classification within case-control pairs using the known, simulated genetic values was 0.67, averaging across 1,000 simulations.

## Discussion

Previous GWAS studies have identified multiple SNPs and genomic locations associated with susceptibility to infection by *MAP* in Holsteins [24], [33], [47], [48], [49], [50] and Jerseys [51]; however, correspondence between the most significant marker loci has been low across studies. Differences in results may reflect differences in case definition, e.g. positive to antibody ELISA [24], [49], [50] versus fecal culture positive [47] versus tissue culture positive [47] or a combination of these [33], [47]. Differences in statistical methodolgies between studies may also have contributed to variation in results. Results from the current study continue that pattern, as no association implicated a region in common with results of previous studies with the exception of a previous analysis of the same data set [33]. In the previous analysis of this data, cases used in the current study were genotyped and allele frequencies compared with allele frequencies derived from sires and maternal grandsires used within the corresponding herds. This case-reference design, akin to a case-parental control analysis [52], identified a significant association on BTA15 (ARS-BFGL-NGS-101744 at 69.1 Mb) within approximately 2.5 Mb of the current result (ARS-BFGL-NGS-110386 at 66.7 Mb). The low correspondence between results from this study and others in independent Holstein populations suggests that it is unlikely that loci with large effect on *MAP* susceptibility exist within the Holstein population or are of such a low frequency that their detection will be difficult within the context of a GWAS analysis. It also suggests the need to combine results from across these studies in a meta-analysis to gain power to identify loci of moderate to small effect. In comparing published results across studies there is an inherent limitation in that only the most significant results are compared. A meta-analysis would provide the opportunity to identify loci with consistent association across studies albeit at lesser significance within any individual study.

Results from the cross-validation analysis suggest low accuracy for the genomic prediction, but this conclusion must be tempered by the recognition that the comparison here is genomic prediction versus a realization of a single event, i.e. did an individual animal manifest evidence of *MAP* infection? Given the low heritability of *MAP* susceptibility phenotypes (∼10%), low accuracy of prediction for a single event is to be expected. Even with the simulation study in which genetic values were known with complete accuracy, probability of correct classification was 0.67. This is directly attributable to the low heritability for the trait, and the value of 0.67 realized in the simulation provides an upper bound for correct classification in this scenario. A more meaningful scenario would be a comparison of the genomic predictions of sires with their average progeny susceptibility based on large numbers (hundreds) of offspring.

Johne's disease in cattle shares similar manifestations to Crohn's disease in humans, thus it is interesting to compare results of the current study with results from GWAS of Crohn's disease in humans. Individual studies and subsequent meta-analyses have identified 131 unique risk loci for Crohn's disease based on the tabulation found at “A Catalog of Published Genome-Wide Association Studies” (http://www.genome.gov/gwastudies/). Comparing the corresponding bovine genomic locations of these Crohn's risk loci with results of the current study suggest two potential overlaps, both on BTA7. SNP ARS-BFGL-NGS-7756 at 71 Mb on BTA7 is approximately 2 Mb from interleukin 12B (IL12B), a subunit of cytokines (interleukins 12 and 23) that activate transcription activator STAT4 and stimulate production of interferon gamma. IL12B has been associated with Crohn's disease in three independent GWAS [53], [54], [55] as well as subsequent candidate gene studies [56], [57], [58]. SNP Hapmap38264-BTA-96587 at 102.4 Mb on BTA7 is 1.6 Mb from solute carrier organic anion transporter family, member 6A1 (SLCO6A1), implicated in a single GWAS of individuals of Ashkenazi Jewish descent [59]. SLCO6A1 was the sole gene in the genomic region identified in that study, though the potential role for this gene in Crohn's disease is unclear. The reported gonad-specific expression of this gene would not seemingly fit with Crohn's disease etiology [60]. While the co-location of the SNPs from this study and these two candidate loci is marginal, both extending over a distance greater than 1 Mb, these loci may merit consideration as positional candidates given that loci exhibiting high linkage disequilibrium can be separated by distances on this order. As previously reported for the US Holstein population, loci with moderate linkage disequilibrium (0.4<r^2^<0.6) were separated by a median distance of ∼1 Mb [61].

An examination of the genomic regions identified in this study suggests potential positional candidate genes in several cases. The 70–71 Mb region of BTA7 was described above as being at a moderate distance (2 Mb) from a gene (IL12B) known to be associated with Crohn's disease in humans. However, additional potential candidate genes are in closer proximity to this region, specifically T-cell immunoglobulin and mucin domain containing 4 (TIMD4) and IL2-inducible T-cell kinase (ITK). TIMD4 is implicated in regulation of T-cell proliferation [62] and ITK encodes an intracellular tyrosine kinase and is thought to affect both T-cell proliferation and differentiation [63], [64]. The gene for complement component 5 (C5) is located approximately 0.7 Mb proximal to the most significant SNP on BTA8 from the GRAMMAR-GC analysis (Table 1). C5 is a strong candidate based on its roles in inflammation and cell killing processes [65]. Finally, the most significant SNP on BTA15 is located approximately 100 kb distal of the gene for the CD44 molecule which is a cell surface protein that is critical for leukocyte migration across the intestinal epitheium [66].

## Supporting Information

Figure S1 **Multi-dimensional scaling plot for the combined discovery and validation data sets.** Case and control samples are indicated as two different colors. PC1 and PC2 are the first two principal components obtained from genomic kinship matrix. Distance between points represents the genetic distance between animals.
(TIF)

Figure S2 **Quantile-quantile plot for results from the GRAMMAR-GC analysis of the combined discovery and validation data.** The Y-axis represents observed P-values and the X-axis represents expected P-values under a null hypothesis (diagonal) of no association.
(TIF)

Dataset S1 **Genotype data for discovery and validation samples.** There are 38,434 genotypes per individual; missing genotypes have been imputed.
(ZIP)

## References

[1] Sweeney RW, Collins MT, Koets AP, McGuirk SM, Roussel AJ (2012) Paratuberculosis (Johne's disease) in cattle and other susceptible species. J Vet Intern Med 26:1239–1250. 23106497 10.1111/j.1939-1676.2012.01019.x

[2] Lombard JE (2011) Epidemiology and economics of paratuberculosis. Vet Clin North Am Food Anim Pract 27:525–535, v. 22023831 10.1016/j.cvfa.2011.07.012

[3] Jubb T, Galvin J (2000) Herd testing to control bovine Johne's disease. Vet Microbiol 77:423–428. 11118727 10.1016/s0378-1135(00)00327-8

[4] Kennedy DJ, Allworth MB (2000) Progress in national control and assurance programs for bovine Johne's disease in Australia. Vet Microbiol 77:443–451. 11118729 10.1016/s0378-1135(00)00329-1

[5] Tharaldsen J, Djonne B, Fredriksen B, Nyberg O, Siguroardottir O (2003) The National Paratuberculosis Program in Norway. Acta Vet Scand 44:243–246. 15074638

[6] Gunnarsson E, Fridriksdottir V, Sigurdarson S (2003) Control of paratuberculosis in Iceland. Acta Vet Scand 44:255. 15074641

[7] Momotani E (2012) Epidemiological situation and control strategies for paratuberculosis in Japan. Jpn J Vet Res 60 Suppl:S19–29. 22458197

[8] Groenendaal H, Nielen M, Hesselink JW (2003) Development of the Dutch Johne's disease control program supported by a simulation model. Prev Vet Med 60:69–90. 12900150 10.1016/s0167-5877(03)00083-7

[9] Carter MA (2011) State, federal, and industry efforts at paratuberculosis control. Vet Clin North Am Food Anim Pract 27:637–645, viii. 22023842 10.1016/j.cvfa.2011.07.010

[10] Nielsen SS, Toft N (2009) A review of prevalences of paratuberculosis in farmed animals in Europe. Prev Vet Med 88:1–14. 18817995 10.1016/j.prevetmed.2008.07.003

[11] National Animal Health Monitoring System (2008) Johne's Disease on U.S. Dairies, 1991–2007.

[12] Dargatz DA, Byrum BA, Hennager SG, Barber LK, Kopral CA, et al (2001) Prevalence of antibodies against *Mycobacterium avium subsp paratuberculosis* among beef cow-calf herds. J Am Vet Med Assoc 219:497–501. 11518178 10.2460/javma.2001.219.497

[13] Lombard JE, Gardner IA, Jafarzadeh SR, Fossler CP, Harris B, et al (2013) Herd-level prevalence of *Mycobacterium avium* subsp. *paratuberculosis* infection in United States dairy herds in 2007. Prev Vet Med 108:234–238. 22979969 10.1016/j.prevetmed.2012.08.006

[14] Ott SL, Wells SJ, Wagner BA (1999) Herd-level economic losses associated with Johne's disease on US dairy operations. Prev Vet Med 40:179–192. 10423773 10.1016/s0167-5877(99)00037-9

[15] Harris NB, Barletta RG (2001) *Mycobacterium avium* subsp. *paratuberculosis* in Veterinary Medicine. Clin Microbiol Rev 14:489–512. 11432810 10.1128/CMR.14.3.489-512.2001 PMC88986

[16] Chiodini RJ, Chamberlin WM, Sarosiek J, McCallum RW (2012) Crohn's disease and the mycobacterioses: a quarter century later. Causation or simple association? Crit Rev Microbiol 38:52–93. 22242906 10.3109/1040841X.2011.638273

[17] Koets AP, Adugna G, Janss LL, van Weering HJ, Kalis CH, et al (2000) Genetic variation of susceptibility to *Mycobacterium avium* subsp. *paratuberculosis* infection in dairy cattle. J Dairy Sci 83:2702–2708. 11104291 10.3168/jds.S0022-0302(00)75164-2

[18] Mortensen H, Nielsen SS, Berg P (2004) Genetic variation and heritability of the antibody response to *Mycobacterium avium* subspecies *paratuberculosis* in Danish Holstein cows. J Dairy Sci 87:2108–2113. 15328223 10.3168/jds.S0022-0302(04)70029-6

[19] Gonda MG, Chang YM, Shook GE, Collins MT, Kirkpatrick BW (2006) Genetic variation of *Mycobacterium avium* ssp. *paratuberculosis* infection in US Holsteins. J Dairy Sci 89:1804–1812. 16606752 10.3168/jds.S0022-0302(06)72249-4

[20] Hinger M, Brandt H, Erhardt G (2008) Heritability estimates for antibody response to *Mycobacterium avium* subspecies *paratuberculosis* in German Holstein cattle. J Dairy Sci 91:3237–3244. 18650301 10.3168/jds.2008-1021

[21] Kupper J, Brandt H, Donat K, Erhardt G (2012) Heritability estimates for *Mycobacterium avium* subspecies *paratuberculosis* status of German Holstein cows tested by fecal culture. J Dairy Sci 95:2734–2739. 22541503 10.3168/jds.2011-4994

[22] van Hulzen KJ, Nielen M, Koets AP, de Jong G, van Arendonk JA, et al (2011) Effect of herd prevalence on heritability estimates of antibody response to *Mycobacterium avium* subspecies *paratuberculosis* . J Dairy Sci 94:992–997. 21257067 10.3168/jds.2010-3472

[23] Zare Y, Shook GE, Collins MT, Kirkpatrick BW (2014) Short communication: Heritability estimates for susceptibility to *Mycobacterium avium* subspecies *paratuberculosis* infection defined by ELISA and fecal culture test results in Jersey cattle. J Dairy Sci 10.3168/jds.2013-7426 24819128

[24] van Hulzen KJ, Schopen GC, van Arendonk JA, Nielen M, Koets AP, et al (2012) Genome-wide association study to identify chromosomal regions associated with antibody response to *Mycobacterium avium* subspecies *paratuberculosis* in milk of Dutch Holstein-Friesians. J Dairy Sci 95:2740–2748. 22541504 10.3168/jds.2011-5005

[25] Shook GE, Chaffer M, Wu XL, Ezra E (2012) Genetic parameters for paratuberculosis infection and effect of infection on production traits in Israeli Holsteins. Anim Genet 43 Suppl 1:56–64. 22742503 10.1111/j.1365-2052.2012.02349.x

[26] Chapman SJ, Hill AV (2012) Human genetic susceptibility to infectious disease. Nat Rev Genet 13:175–188. 22310894 10.1038/nrg3114

[27] Brachi B, Morris GP, Borevitz JO (2011) Genome-wide association studies in plants: the missing heritability is in the field. Genome Biol 12:232. 22035733 10.1186/gb-2011-12-10-232 PMC3333769

[28] Finlay EK, Berry DP, Wickham B, Gormley EP, Bradley DG (2012) A genome wide association scan of bovine tuberculosis susceptibility in Holstein-Friesian dairy cattle. PLoS One 7:e30545. 22355315 10.1371/journal.pone.0030545 PMC3280253

[29] Collins MT, Eggleston V, Manning EJ (2010) Successful control of Johne's disease in nine dairy herds: results of a six-year field trial. J Dairy Sci 93:1638–1643. 20338441 10.3168/jds.2009-2664

[30] Collins MT, Kenefick KB, Sockett DC, Lambrecht RS, McDonald J, et al (1990) Enhanced radiometric detection of *Mycobacterium paratuberculosis* by using filter-concentrated bovine fecal specimens. J Clin Microbiol 28:2514–2519. 2254428 10.1128/jcm.28.11.2514-2519.1990 PMC268217

[31] Collins MT (2002) Interpretation of a commercial bovine paratuberculosis enzyme-linked immunosorbent assay by using likelihood ratios. Clin Diagn Lab Immunol 9:1367–1371. 12414776 10.1128/CDLI.9.6.1367-1371.2002 PMC130105

[32] Collins MT, Gardner IA, Garry FB, Roussel AJ, Wells SJ (2006) Consensus recommendations on diagnostic testing for the detection of paratuberculosis in cattle in the United States. J Am Vet Med Assoc 229:1912–1919. 17173528 10.2460/javma.229.12.1912

[33] Kirkpatrick BW, Shi X, Shook GE, Collins MT (2010) Whole-Genome association analysis of susceptibility to paratuberculosis in Holstein cattle. Anim Genet 10.1111/j.1365-2052.2010.02097.x 20618184

[34] Purcell S, Neale B, Todd-Brown K, Thomas L, Ferreira MA, et al (2007) PLINK: a tool set for whole-genome association and population-based linkage analyses. Am J Hum Genet 81:559–575. 17701901 10.1086/519795 PMC1950838

[35] Browning BL, Browning SR (2009) A unified approach to genotype imputation and haplotype-phase inference for large data sets of trios and unrelated individuals. Am J Hum Genet 84:210–223. 19200528 10.1016/j.ajhg.2009.01.005 PMC2668004

[36] Aulchenko YS, Ripke S, Isaacs A, van Duijn CM (2007) GenABEL: an R library for genome-wide association analysis. Bioinformatics 23:1294–1296. 17384015 10.1093/bioinformatics/btm108

[37] R_Core_Team (2013) R: A language and environment for statistical computing. Vienna, Austria: R Foundation for Statistical Computing.

[38] Amin N, van Duijn CM, Aulchenko YS (2007) A genomic background based method for association analysis in related individuals. PLoS One 2:e1274. 18060068 10.1371/journal.pone.0001274 PMC2093991

[39] Aulchenko YS, de Koning DJ, Haley C (2007) Genomewide rapid association using mixed model and regression: a fast and simple method for genomewide pedigree-based quantitative trait loci association analysis. Genetics 177:577–585. 17660554 10.1534/genetics.107.075614 PMC2013682

[40] Benjamini Y, Hochberg Y (1995) Controlling the False Discovery Rate - a Practical and Powerful Approach to Multiple Testing. Journal of the Royal Statistical Society Series B-Methodological 57:289–300.

[41] Fernando RL, Garrick DJ (2008) GenSel - User manual for a portfolio of genomic selection related analyses. Animal Breeding and Genetics, Iowa State University, Ames.

[42] Meuwissen TH, Hayes BJ, Goddard ME (2001) Prediction of total genetic value using genome-wide dense marker maps. Genetics 157:1819–1829. 11290733 10.1093/genetics/157.4.1819 PMC1461589

[43] Kizilkaya K, Fernando RL, Garrick DJ (2010) Genomic prediction of simulated multibreed and purebred performance using observed fifty thousand single nucleotide polymorphism genotypes. J Anim Sci 88:544–551. 19820059 10.2527/jas.2009-2064

[44] Onteru SK, Fan B, Du ZQ, Garrick DJ, Stalder KJ, et al (2012) A whole-genome association study for pig reproductive traits. Anim Genet 43:18–26. 22221021 10.1111/j.1365-2052.2011.02213.x

[45] Salzberg SL (1997) On Comparing Classifiers: Pitfalls to Avoid and a Recommended Approach. New York, NY: Springer. 11 p.

[46] Sing T, Sander O, Beerenwinkel N, Lengauer T (2005) ROCR: visualizing classifier performance in R. Bioinformatics 21:3940–3941. 16096348 10.1093/bioinformatics/bti623

[47] Settles M, Zanella R, McKay SD, Schnabel RD, Taylor JF, et al (2009) A whole genome association analysis identifies loci associated with *Mycobacterium avium* subsp. *paratuberculosis* infection status in US Holstein cattle. Anim Genet 40:655–662. 19422364 10.1111/j.1365-2052.2009.01896.x

[48] Neibergs HL, Settles ML, Whitlock RH, Taylor JF (2010) GSEA-SNP identifies genes associated with Johne's disease in cattle. Mamm Genome 21:419–425. 20706723 10.1007/s00335-010-9278-2

[49] Minozzi G, Buggiotti L, Stella A, Strozzi F, Luini M, et al (2010) Genetic loci involved in antibody response to *Mycobacterium avium* ssp. *paratuberculosis* in cattle. PLoS One 5:e11117. 20559561 10.1371/journal.pone.0011117 PMC2886106

[50] Pant SD, Schenkel FS, Verschoor CP, You Q, Kelton DF, et al (2010) A principal component regression based genome wide analysis approach reveals the presence of a novel QTL on BTA7 for MAP resistance in holstein cattle. Genomics 95:176–182. 20060464 10.1016/j.ygeno.2010.01.001

[51] Zare Y, Shook GE, Collins MT, Kirkpatrick BW (2014) Genome-wide association analysis and genomic prediction of *Mycobacterium avium* subspecies *paratuberculosis* infection in US Jersey cattle. PLoS One 9:e88380. 24523889 10.1371/journal.pone.0088380 PMC3921184

[52] Flanders WD, Khoury MJ (1996) Analysis of case-parental control studies: method for the study of associations between disease and genetic markers. Am J Epidemiol 144:696–703. 8823066 10.1093/oxfordjournals.aje.a008982

[53] Barrett JC, Hansoul S, Nicolae DL, Cho JH, Duerr RH, et al (2008) Genome-wide association defines more than 30 distinct susceptibility loci for Crohn's disease. Nat Genet 40:955–962. 18587394 10.1038/NG.175 PMC2574810

[54] McGovern DP, Jones MR, Taylor KD, Marciante K, Yan X, et al (2010) Fucosyltransferase 2 (FUT2) non-secretor status is associated with Crohn's disease. Hum Mol Genet 19:3468–3476. 20570966 10.1093/hmg/ddq248 PMC2916706

[55] Parkes M, Barrett JC, Prescott NJ, Tremelling M, Anderson CA, et al (2007) Sequence variants in the autophagy gene IRGM and multiple other replicating loci contribute to Crohn's disease susceptibility. Nat Genet 39:830–832. 17554261 10.1038/ng2061 PMC2628541

[56] Moon CM, Shin DJ, Son NH, Shin ES, Hong SP, et al (2013) Genetic variants in the IL12B gene are associated with inflammatory bowel diseases in the Korean population. J Gastroenterol Hepatol 28:1588–1594. 23573954 10.1111/jgh.12214

[57] Glas J, Seiderer J, Wagner J, Olszak T, Fries C, et al (2012) Analysis of IL12B gene variants in inflammatory bowel disease. PLoS One 7:e34349. 22479607 10.1371/journal.pone.0034349 PMC3316707

[58] Yamazaki K, Takahashi A, Takazoe M, Kubo M, Onouchi Y, et al (2009) Positive association of genetic variants in the upstream region of NKX2-3 with Crohn's disease in Japanese patients. Gut 58:228–232. 18936107 10.1136/gut.2007.140764

[59] Kenny EE, Pe'er I, Karban A, Ozelius L, Mitchell AA, et al (2012) A genome-wide scan of Ashkenazi Jewish Crohn's disease suggests novel susceptibility loci. PLoS Genet 8:e1002559. 22412388 10.1371/journal.pgen.1002559 PMC3297573

[60] Lee SY, Williamson B, Caballero OL, Chen YT, Scanlan MJ, et al (2004) Identification of the gonad-specific anion transporter SLCO6A1 as a cancer/testis (CT) antigen expressed in human lung cancer. Cancer Immun 4:13. 15546177

[61] Kim ES, Kirkpatrick BW (2009) Linkage disequilibrium in the North American Holstein population. Anim Genet 40:279–288. 19220233 10.1111/j.1365-2052.2008.01831.x

[62] Meyers JH, Chakravarti S, Schlesinger D, Illes Z, Waldner H, et al (2005) TIM-4 is the ligand for TIM-1, and the TIM-1-TIM-4 interaction regulates T cell proliferation. Nat Immunol 6:455–464. 15793576 10.1038/ni1185

[63] Kosaka Y, Felices M, Berg LJ (2006) Itk and Th2 responses: action but no reaction. Trends Immunol 27:453–460. 16931156 10.1016/j.it.2006.08.006

[64] Gomez-Rodriguez J, Kraus ZJ, Schwartzberg PL (2011) Tec family kinases Itk and Rlk/Txk in T lymphocytes: cross-regulation of cytokine production and T-cell fates. FEBS J 278:1980–1989. 21362139 10.1111/j.1742-4658.2011.08072.x PMC3117960

[65] Elmgreen J (1984) Subnormal activation of phagocytes by complement in chronic inflammatory bowel disease? Neutrophil chemotaxis to complement split product C5a. Gut 25:737–742. 6735255 10.1136/gut.25.7.737 PMC1432611

[66] Brazil JC, Liu R, Sumagin R, Kolegraff KN, Nusrat A, et al (2013) alpha3/4 Fucosyltransferase 3-dependent synthesis of Sialyl Lewis A on CD44 variant containing exon 6 mediates polymorphonuclear leukocyte detachment from intestinal epithelium during transepithelial migration. J Immunol 191:4804–4817. 24068663 10.4049/jimmunol.1301307 PMC4047976

