# Supplementary figures and images for: Genome-Wide Association Study of Susceptibility to Infection by Mycobacterium avium Subspecies paratuberculosis in Holstein Cattle
Source: PLoS One. 2014 Dec 4;9(12):e111704. doi: 10.1371/journal.pone.0111704 (PMC4256300; doi:10.1371/journal.pone.0111704)

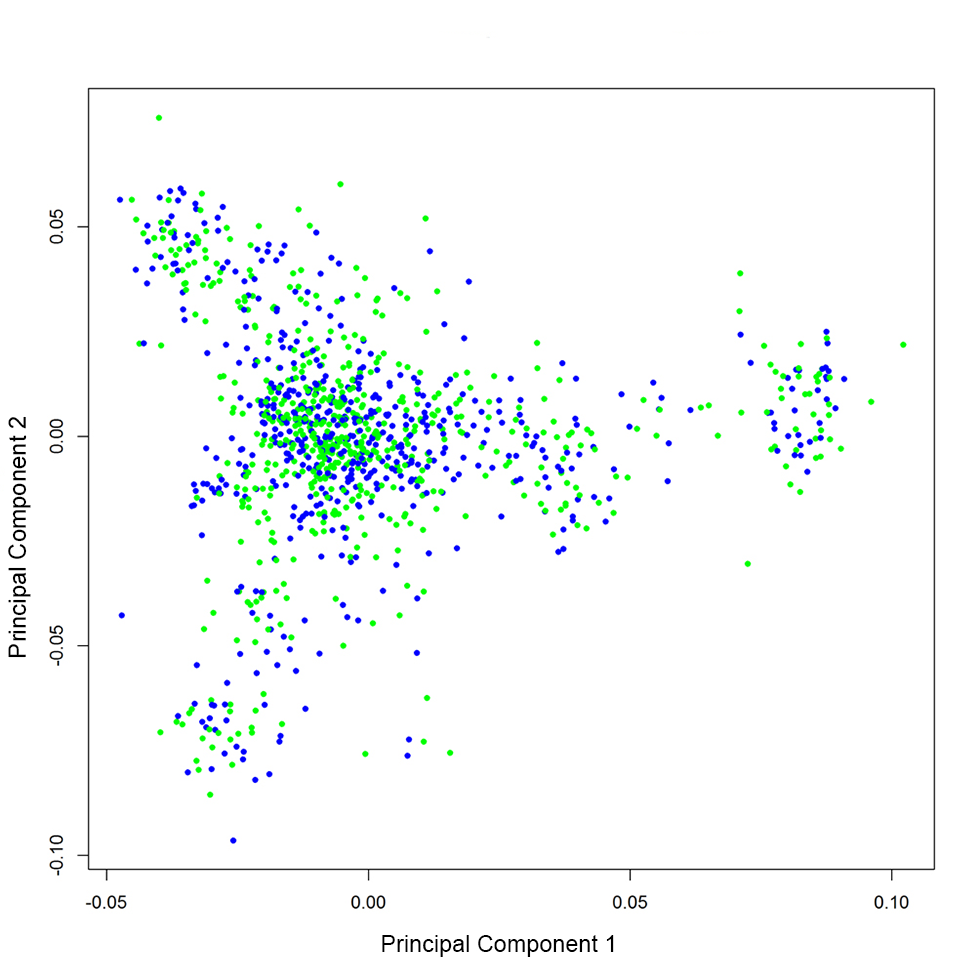

Supplement: Figure S1 — Multi-dimensional scaling plot for the combined discovery and validation data sets. Case and control samples are indicated as two different colors. PC1 and PC2 are the first two principal components obtained from genomic kinship matrix. Distance between points represents the genetic distance between animals. (TIF) [file pone.0111704.s001.tif]

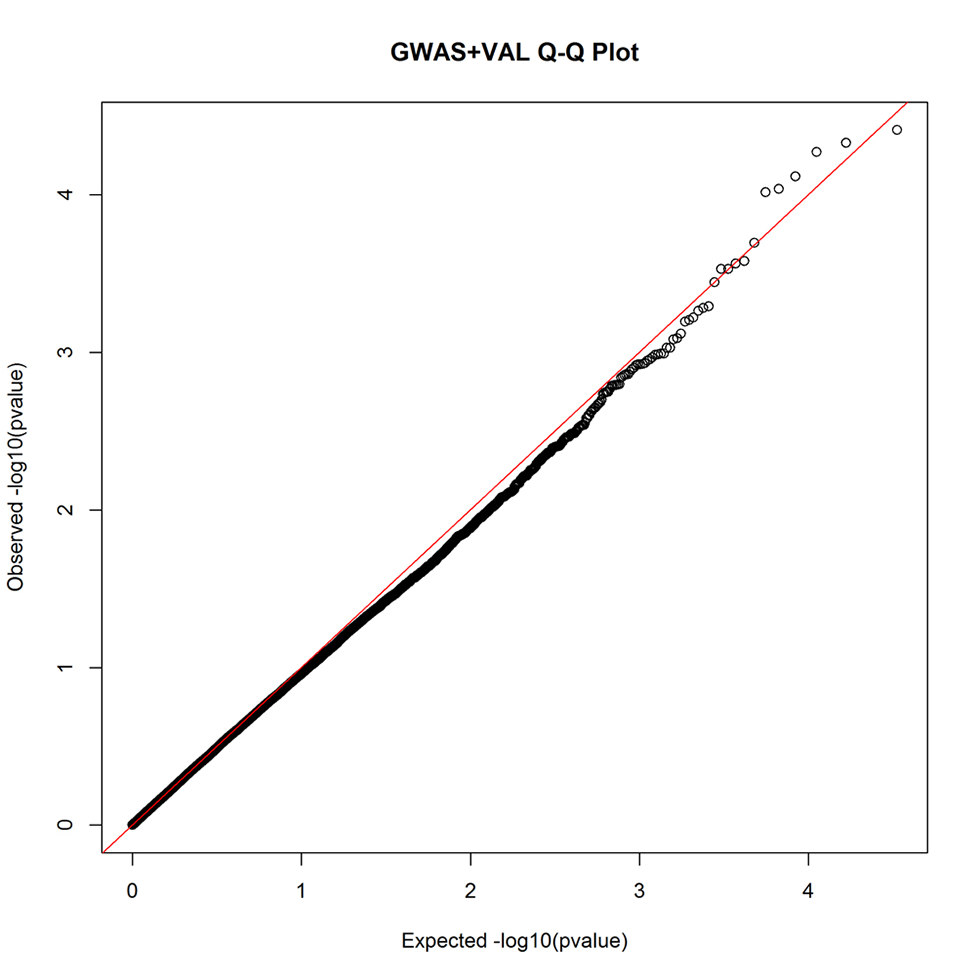

Supplement: Figure S2 — Quantile-quantile plot for results from the GRAMMAR-GC analysis of the combined discovery and validation data. The Y-axis represents observed P-values and the X-axis represents expected P-values under a null hypothesis (diagonal) of no association. (TIF) [file pone.0111704.s002.tif]
